# Green Synthesis of Silver Nanoparticles Using Cashew Nutshell Liquid (CNSL): Characterization and Methylene Blue Removal Studies

**DOI:** 10.3390/molecules29163895

**Published:** 2024-08-17

**Authors:** Justyn Carollo, Daniel Ballesteros-Plata, Elena Rodríguez-Aguado, Svetlana Bashkova

**Affiliations:** 1 Department of Chemistry, Biochemistry, and Physics, Fairleigh Dickinson University, Madison, NJ 07940, USA; jcarollo@student.fdu.edu; 2 Departamento de Química Inorgánica, Facultad de Ciencias, Universidad de Málaga, Campus de Teatinos, 29071 Málaga, Spain; daniel.ballesteros@uma.es (D.B.-P.); aguadoelena5@gmail.com (E.R.-A.)

**Keywords:** silver nanoparticles, cashew nutshell liquid, dye removal, methylene blue, catalytic reduction, green synthesis

## Abstract

In this work, silver nanoparticles (AgNPs) were synthesized from cashew nutshell liquid (CNSL) by varying the concentration of silver ions and the pH of the CNSL extract. The synthesized AgNPs were further characterized to study their surface, structural, and morphological properties and tested for the removal of methylene blue (MB) dye. The results of this study showed that depending on the conditions, particles of various sizes, ranging from 1 to 60 nm, and different degrees of stabilization and agglomeration were produced. The concentration of silver ions equal to 3 mM and the pH of the extract of ~4.5 (AgNP3) resulted in the most efficient synthesis, where particles appeared to be highly stabilized and homogeneously distributed on the surface, exhibiting a small average particle size and a narrow particle size distribution (6.7 ± 6.5 nm). Such particles further showed the highest percent removal of MB, where up to 80% removal was recorded within the first 20 min. Higher concentrations of silver ions and higher pH of the extract resulted in substantial particle agglomeration and particles being over-capped by the CNSL biomolecules, respectively, which further negatively affected the ability of particles to remove MB. Finally, the fact that visible light showed no significant effect on the removal of MB, with the average removal rates found to be about the same as in the dark, suggests the strong catalytic nature of AgNPs, which facilitates the electron transfer reactions leading to MB reduction.

## 1. Introduction

Silver nanoparticles (AgNPs) are well known for their anti-bacterial, anti-fungal, anti-viral, and degradation properties attributed to their small size of less than 100 nm and their high surface area to volume ratio [1,2,3,4,5,6,7,8,9,10]. AgNPs are very effective and safe at low concentrations, as they have shown higher antibacterial activity than penicillin, biomycin, and other antibiotics, with low cytotoxicity and immunological response [3,4,7,8], as well as being capable of preventing the growth of bacteria like *Bacillus cereus*, *Staphylococcus aureus* (*S. aureus*), *Escherichia coli* (*E. coli*), *Citrobacter koseri*, and many more [1,4,7,11]. In addition, the functional groups on the surface of AgNPs provide electrostatic interactions with multiple pollutant molecules, assisting in their removal and degradation [12,13,14,15]. Considering the above-mentioned properties of silver nanoparticles, some of their common applications include water purification, drug delivery, medical imaging, molecular diagnostics, therapeutics, food processing, and food packaging [4,5,7,9,10,12,16]. 

While silver nanoparticles have been proven beneficial, mass synthesizing them in a regulated manner with an efficient and eco-friendly process remains challenging [6,7,9,10]. Most metallic nanoparticles are made from a “bottom-up” approach, which involves having atoms group up together to form the nanoparticles [1,7,10,17,18]. The benefit of this technique is that the reducing agent can control the particles’ structures and act as the catalyst for the synthesis [1,5,7,8,16]. Furthermore, biological methods are preferred over chemical and physical methods, as the former are more cost-effective, energy-efficient, simple, eco-friendly, and can be scaled up to produce high amounts of nanoparticles [1,5,6,7,8,9,16,18]. In green synthesis using plants, biomolecules and secondary metabolites such as flavonoids, ketones, aldehydes, tannins, and carboxylic acids act as reducing and stabilizing agents to convert silver ions to elemental silver [1,5,6,7,9,10,16,19]. First, the biomolecules’ antioxidant functional groups donate electrons to the silver ions to produce silver atoms that group up and nucleate to form nanoparticles of a specific size [7,10,17,19,20]. Lastly, the phytochemicals cap or stabilize the nanoparticles by binding to them, with the polar head of a phytomolecule coordinated towards the metal atom and the nonpolar tail encircling the medium, often enabling a synergistic effect between the AgNP and extract’s properties [7,10,17,19,20].

Different studies have shown that the concentration of the silver ions, the temperature, the pH of the solution, and the reaction time influence the size, shape, and morphology of the AgNPs, which in turn impact their properties [3,5,6,7,16,17,20]. Thus, when changing the precursor concentration, higher amounts will often bring about a greater yield and smaller particles, but too much usually causes the size of the nanoparticles to increase [6,7,17]. Furthermore, temperatures between 25 °C and 75 °C have been found to produce more spherical nanoparticles and to boost the speed of the synthesis [5,6,16,17,21], but temperatures above 75 °C were determined to disfavor the reduction process of silver ions due to the degradation of the biomolecules responsible for AgNP synthesis and particle aggregation. Moreover, basic media have been proven to be beneficial for the stability of nanoparticle synthesis due to the biomass’s electrical charge being more suitable for the binding, reduction, and stabilization of the metal ions [6,7,10,16,17]. Finally, optimizing the period of the synthesis is necessary for stability, so that the nanoparticles do not cluster into a bulk metal due to agglomeration [6,7,16,17]. Close control of the above-mentioned parameters can be challenging at times as aggregation and other factors may have an unexpected effect on the physical properties of the nanoparticles [7,17].

In this work, a green approach is applied to prepare silver nanoparticles with cashew nutshell liquid (CNSL) as a reducing agent and by varying the synthesis conditions to control the properties of AgNPs. In CNSL, secondary metabolites involved in nanoparticle formation are non-isoprenoid lipids including anacardic acids, cardols, cardanols, methylcardols, and polymeric materials [22,23,24]. Here cardanols and cardols are expected to play a pivotal role in the silver nanoparticle formation as they have been proven to strongly govern the antioxidant properties and the ability of CNSL to neutralize free radicals by giving up electrons [23,25].

The produced AgNPs are further tested for their dye-removal properties. For dye removal, methylene blue—cationic azo dye—is chosen as a pollutant of interest. Dye pollution has become more abundant in our water systems due to discharge from paper, textile, leather, food, cosmetic, and pharmaceutical industries [2,26,27,28]. Out of the roughly 2.1 metric tons of dye produced worldwide, about 15% enter our waterways, effectively decreasing the water’s transparency, which causes less light to reach aquatic plants [26]. This hinders photosynthesis, lowering the amount of dissolved oxygen in water, which is essential for aquatic ecosystems to support life [26,29]. Additionally, synthetic dyes are non-biodegradable and release possible carcinogenic products [27,28,29], which makes their removal imperative for healthy water systems. 

## 2. Results

### 2.1. Formation Rates of AgNPs

**Figure 1:**
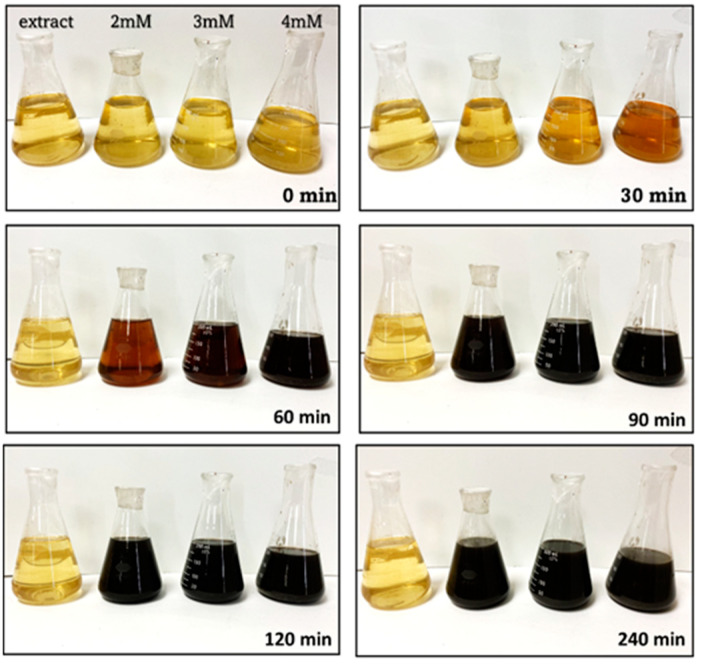
The change in solution color over time during the AgNP synthesis at concentrations of 2, 3, and 4 mM AgNO_3_ and a pH of the extract of 4.5 in comparison to that of a pure CNSL extract at pH 4.5.

**Figure 2:**
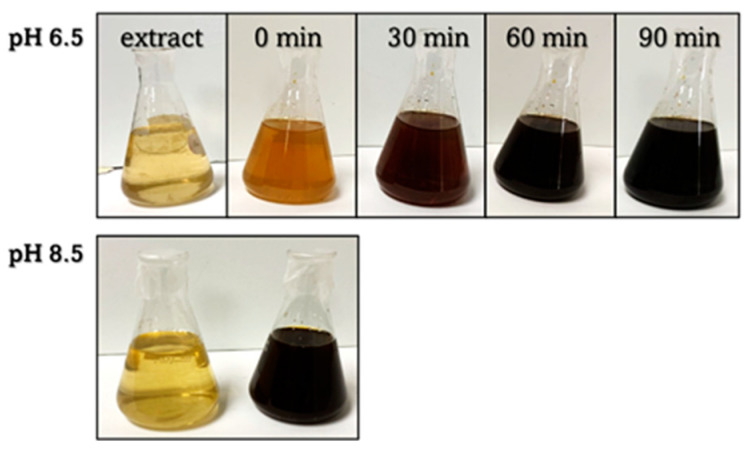
The change in solution color over time during the AgNP synthesis at a pH of the extract of 6.5 and 8.5 and 3 mM AgNO_3_ in comparison to that of a pure extract at pH of 6.5 and 8.5.

Figure 1 and Figure 2 demonstrate how varying the concentration of AgNO_3_ and the pH of the extract altered the formation rates of the AgNPs, where a quicker color change from yellow to black indicates an increased reaction rate. The formation of AgNPs took place within 60–90 min for different concentrations of AgNO_3_ (Figure 1) and within ~30 min at the higher pH values of the extract (Figure 2); the increase in the formation rate was observed with the increase in the concentration of AgNO_3_ and the pH of the extract.

**Figure 3:**
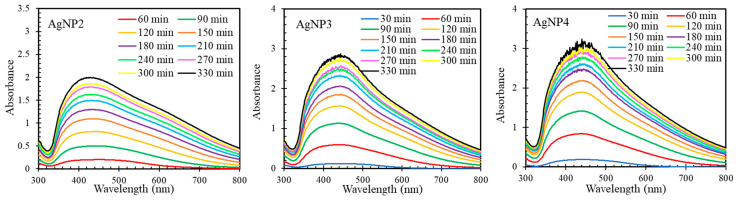
The AgNP surface plasmon resonance curves during the synthesis with 2, 3, and 4 mM AgNO_3_ and a pH of the extract of 4.5.

Figure 3 shows the resulting surface plasmon resonance curves for AgNPs synthesized from different concentrations of AgNO_3_. For each concentration, a single SPR peak was produced, and according to Mie’s theory, this indicates that the AgNPs are spherical rather than anisotropic in shape [6,11]. The curves became more intense as the synthesis proceeded and as the concentration of AgNO_3_ increased. More so, at higher concentrations of AgNO_3_, the curves became sharper and more intense during a shorter interval of time. These results indicate that solutions with more available silver cations made the reduction process more favorable [2,6,7,8,9,30,31]. The change in AgNP properties was most noticeable when increasing the AgNO_3_ concentration from 2 mM to 3 mM but was much less prominent from 3 mM to 4 mM. Additionally, a red shift occurred from 424 nm for AgNP2 to 442 nm for AgNP3 and AgNP4, likely from electron delocalization, suggesting a change in AgNP size [2,6,8,11,32,33].

**Figure 4:**
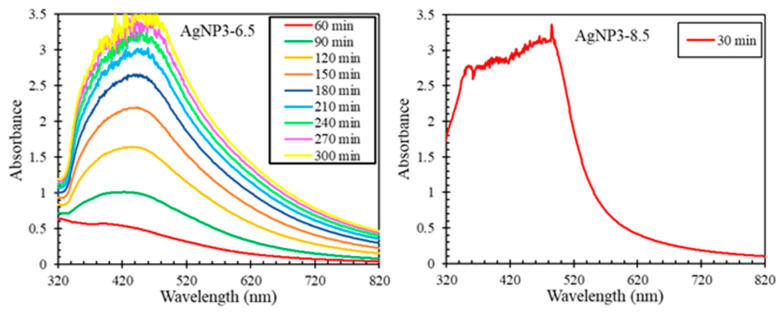
The AgNP surface plasmon resonance curves during the synthesis with a pH of the extract of 6.5 and 8.5 and an AgNO_3_ concentration of 3 mM.

For AgNPs synthesized at a higher pH of the extract, the formation rate of nanoparticles increased substantially at pH 6.5 and was instant at pH 8.5 (Figure 4). These results agree with the color observations shown in Figure 2. The faster reaction rate of the synthesis in a more basic medium could be attributed to the increased negative electrical charge of the biomolecules’ functional groups to reduce, cap, and stabilize the AgNPs [6,7,10,11].

### 2.2. MB Removal Studies

**Figure 5:**
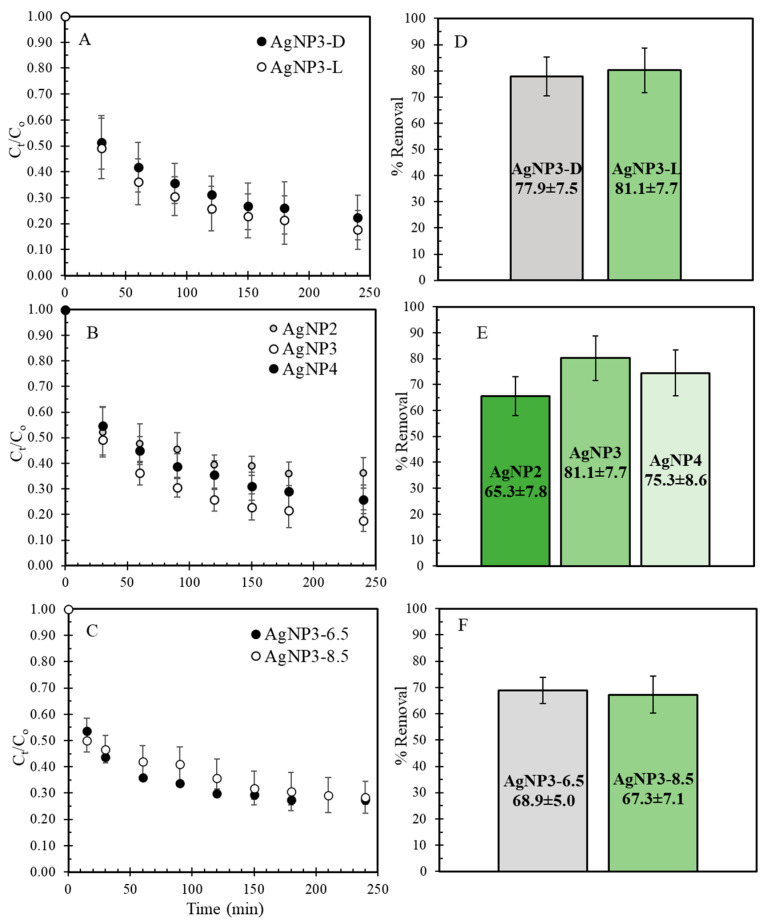
The MB discoloration curves and comparison in percent removal for (**A**,**D**) AgNPs synthesized at 3mM AgNO_3_ and a pH of the extract of 4.5 in the dark and under green light; (**B**,**E**) for AgNPs synthesized at 2, 3, and 4 mM AgNO_3_ and a pH of the extract of 4.5 under green light; (**C**,**F**) for AgNPs synthesized at 3 mM AgNO_3_ and pH of the extract of 6.5 and 8.5 under green light.

The synthesized AgNPs were tested for MB removal in the dark and under green light to study the effect of visible light on the extent of removal. In the case of the latter, the light energy could promote the AgNP electrons from the valance to the conduction band, resulting in the formation of electron–hole pairs that are highly reactive and could trigger electron transfers with the MB molecules, causing their photodegradation [11,29,33,34,35]. The results of this MB removal study are shown in Figure 5A. Here, only a slight increase in the average percent removal is observed under green light, with no major improvement in the performance of AgNPs. Thus, the average percentage removal across five or more different batches is about 3% higher under green light than in the dark (Figure 5D). Furthermore, AgNPs synthesized from different concentrations of AgNO_3_ were tested and compared under green light. Figure 5B,E show that the extent of removal was, on average, the highest for AgNP3, followed by AgNP4 (decrease in % removal of ~6%) and AgNP2 (decrease in % removal of ~16%). These results indicate that varying the concentration of AgNO_3_ produced nanoparticles of different properties, which in turn affected their performance for the removal of MB. As per AgNPs synthesized at a higher pH of the extract, the average % removal decreased by ~15% for AgNP3-6.5 and by ~17% for AgNP3-8.5 (Figure 5C,F) in comparison to AgNP3 synthesized at pH 4.5. This indicates that although increasing the pH of the extract should be beneficial for the stability of nanoparticle synthesis due to the enhanced electrostatic interactions between CNSL and silver ions, samples produced at higher pH did not show any improvement in MB removal. 

### 2.3. XRD Analysis

**Figure 6:**
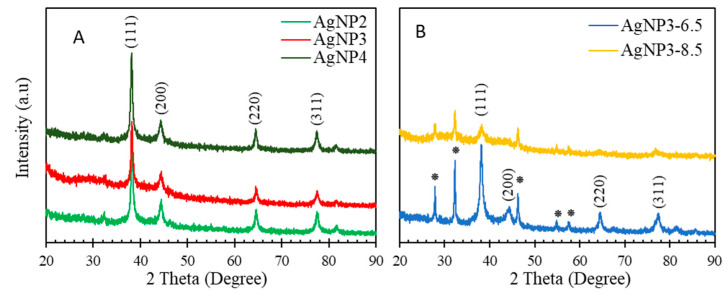
XRD patterns of (**A**) AgNPs synthesized at 2, 3, and 4 mM AgNO_3_ and the pH of the extract of 4.5 and (**B**) AgNPs synthesized at the pH of the extract of 6.5 and 8.5 and the concentration of AgNO_3_ of 3 mM. Peak reflections denoted by (*) are attributed to either the amorphous organic phase or silver chloride.

The crystalline structure of AgNPs was studied by XRD in the range of 2*θ* Bragg angles of 5 to 90°. The XRD diffractograms of AgNPs are presented in Figure 6. Here, the clear reflections at 38.1° (111), 44.4° (200), 64.6° (220), and 77.5° (311) are characteristic of the face-centered cubic silver [JCPDF Card No-087–0720]. The additional peaks at 27.9°, 32.3°, 46.3°, 54.9°, and 57.7° denoted by (*) in Figure 6B are observed for AgNP3-6.5 and AgNP3-8.5 and could be attributed to either the amorphous organic phase [36] or silver chloride [37]. The latter is likely due to the pH adjustments of AgNP3-6.5 and AgNP3-8.5 with hydrochloric acid.

The average crystalline size of silver nanoparticles was calculated from the Debye–Sherrer equation using the reflections at 38.1°, 44.4°, 64.6°, and 77.5°, which were found to be ~16 nm for AgNP2, ~12 nm for AgNP3, ~17 nm AgNP4, and ~11 nm for AgNP3-6.5. The intensities of the reflections for AgNP3-8.5 were too low to estimate the particle size. 

### 2.4. XPS Analysis

**Figure 7:**
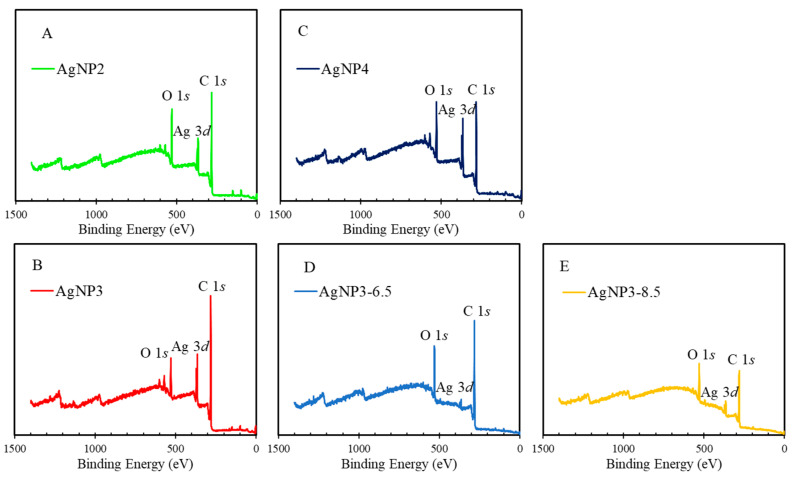
XPS surveys for (**A**) AgNPs synthesized at 2 mM AgNO_3_ and a pH of the extract of 4.5; (**B**) AgNPs synthesized at 3 mM AgNO_3_ and a pH of the extract of 4.5; (**C**) AgNPs synthesized at 4 mM AgNO_3_ and a pH of the extract of 4.5; (**D**) AgNP synthesized at 3 mM AgNO_3_ and a pH of the extract of 6.5; (**E**) AgNP synthesized at 3 mM AgNO_3_ and a pH of the extract of 8.5.

**Figure 8:**
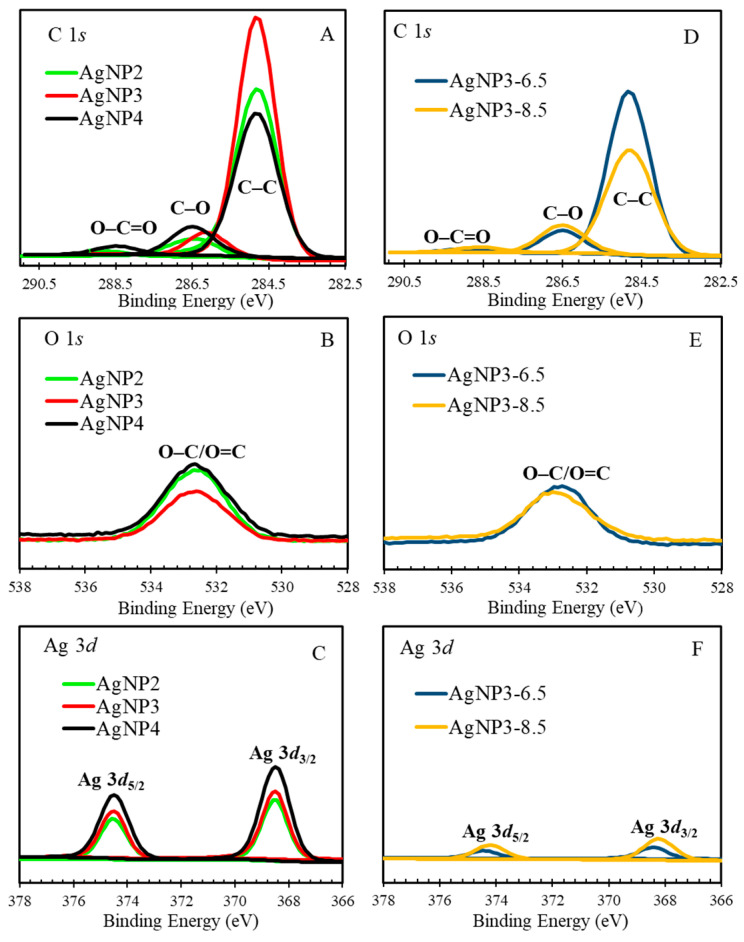
(**A**) C 1*s*, (**B**) O 1*s*, (**C**) Ag 3*d* core-level XPS spectra of AgNPs synthesized at 2, 3, and 4 mM AgNO_3_ at the pH of the extract of 4.5; (**D**) C 1*s*, (**E**) O 1*s*, (**F**) Ag 3*d* core-level XPS spectra of AgNPs synthesized at the pH of the extract of 6.5 and 8.5 and the concentration of AgNO_3_ of 3 mM.

XPS analysis studied the surface chemical composition, and the results of this study are presented in Table 1 and Figure 7 and Figure 8. For reference, CNSL obtained by boiling the shells in water is expected to be similar in composition to natural CNSL, which contains ~60–65% anacardic acid, ~10% cardanol, ~11% cardol, and ~2% 2-methyl cardol [38]. Accordingly, the deconvolution of the C 1*s* core-level spectrum revealed the presence of C–C, C–O, and O–C=O functionalities, corresponding to the presence of the above-mentioned components. Here, C–C linkages related to carbon sp^3^ hybridization could be associated with the nonpolar hydrocarbon ends of CNSL extract components [22,23,24,39,40]. On the other hand, C–O and O–C=O functionalities, related to phenol and carboxylic groups, respectively [23,39,40], could be linked to the polar ends. In addition, the O 1*s* peak at 532.6 eV further suggests that most of the polar surface functionalities are of O–C type [41]. Noteworthily, AgNP3 contains the highest amount of C–C bonds (C 1*s* peak at 284.8 eV) and the lowest amount of surface oxygen-containing groups; no carboxylic functionalities (anacardic acid) were detected, and the C 1*s* peak at 286.5 eV (for AgNP2 and AgNP4) shifted to 286.1 eV (Figure 8A,C and Table 1). These findings suggest that the capping and stabilization process for AgNP3 was the most successful, with the polar heads (C–O and O–C=O groups) of CNSL extract coordinated towards the bulk and facilitating the reduction of Ag^+^ to Ag^0^ and the nonpolar tails (C–C) encircling and stabilizing the nanoparticle medium. Furthermore, the analysis of the Ag 3*d* core-level spectrum reveals the presence of two main contributions ascribed to the doublet Ag 3*d*_5/2_ and Ag 3*d*_3/2_, which, respectively, appear at 368.5 and 374.5 eV and are attributed to metallic silver [42,43].

### 2.5. HRTEM/STEM-EDX Analysis

**Figure 9:**
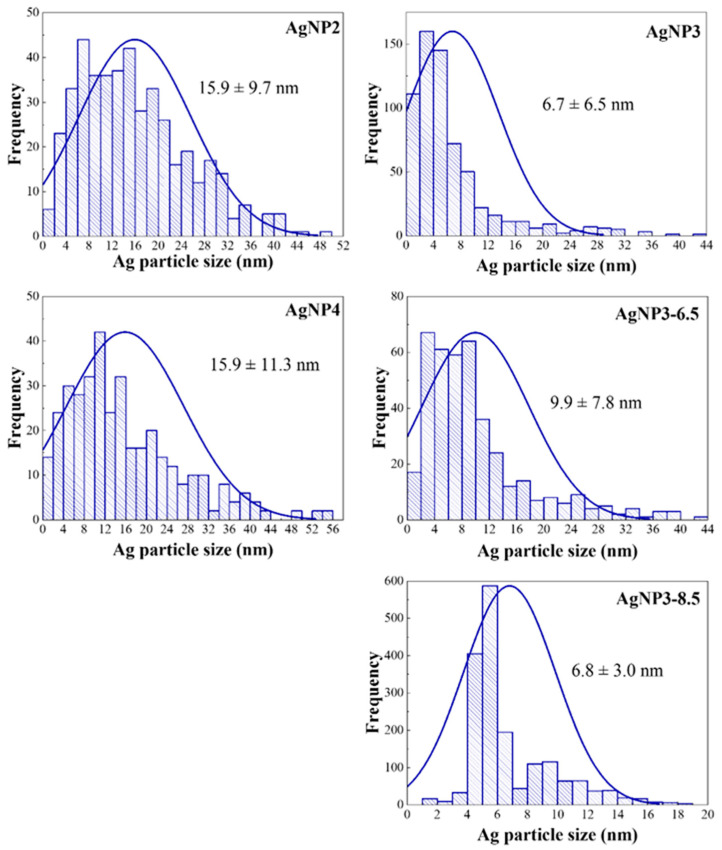
Particle size distribution for AgNP samples obtained from EDX mapping.

The morphology of the synthesized AgNPs was studied by HRTEM and STEM-EDX topography. The micrographic analysis of the AgNP samples revealed that they contain silver particles of different sizes, with particles of a considerable size found in all the samples except AgNP3-8.5 (Table 2, Figure 9). More so, it is observed that the AgNP3 samples have the smallest silver particle sizes compared to the AgNP2 and AgNP4 samples. The AgNP3 samples also have silver particles with a more uniform size, particularly AgNP3-8.5, which has the narrowest silver particle size range.

**Figure 10:**
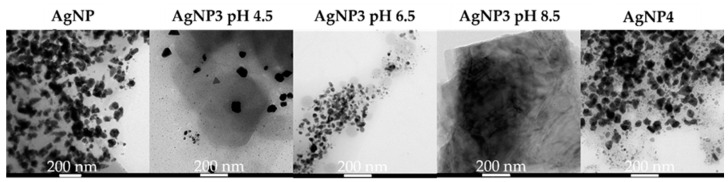
Micrographs of AgNPs obtained by HRTEM.

**Figure 11:**
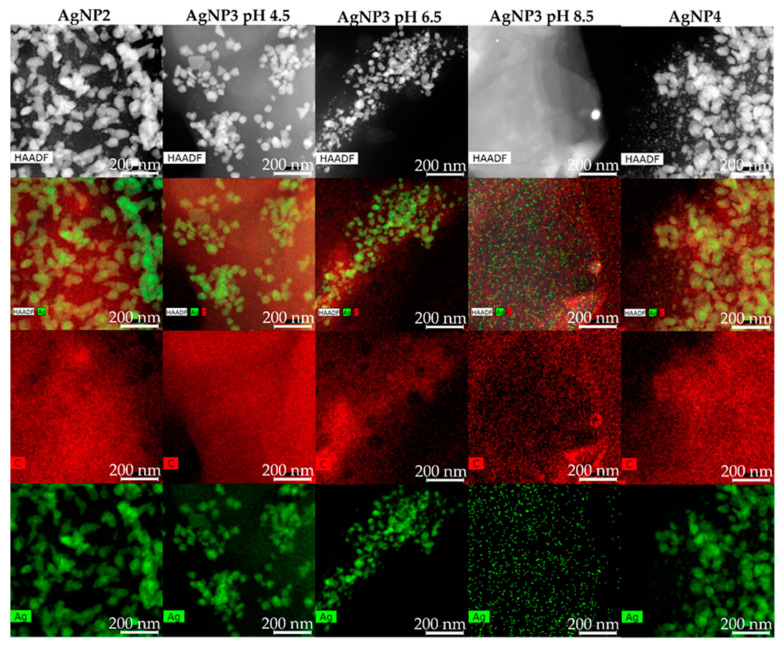
Maps of AgNPs obtained from STEM-EDX analysis.

Furthermore, micrographs obtained by HRTEM (Figure 10) and the mapping from EDX microanalysis (Figure 11) revealed that silver particles are homogeneously distributed on the carbonaceous support of all the samples, with the most uniform and homogeneous distribution of particles found for AgNP3-8.5. All the other samples exhibited a degree of particle agglomeration, with the most pronounced found in the AgNP2 and AgNP4 samples, where some particles reached up to 50 nm in size.

## 3. Discussion

Noteworthily, the fact that the concentration of MB blue is significantly reduced upon contact with the particles’ surface even in the absence of light indicates the strong catalytic nature of AgNPs. It is likely that the primary removal process here starts with the adsorption of MB cationic dye on the negatively charged sites of AgNPs, followed by electron transfer reactions, facilitated by the stabilizing and reducing agent (CNSL extract), which promote the reduction of MB. As reported by multiple authors [44,45,46], the most likely product of this reduction process is a colorless leucomethylene blue (LMB). In addition, the presence of oxygen can also increase the rate of MB reduction by generating reactive oxygen species (ROS), even in the dark [47]. Furthermore, a slight increase in the removal of MB upon exposure to light is likely due to the additional formation of ROS in these conditions.

Among the samples studied, AgNP3 had on average the best performance, suggesting that the properties of AgNP3 nanoparticles were somewhat superior for the catalytic reduction of MB. Referring to the results of XPS analysis, the capping and stabilization process for AgNP3 was the most successful, where there seemed to be enough readily available silver cations to be reduced along with the necessary number of biomolecules to cap and stabilize the AgNPs effectively. In addition, its homogeneous and narrow particle size distribution with a minor degree of agglomeration, small average particle size, and the highest number of particles less than 8 nm in size (Figure 9) further proved to be beneficial for the catalytic reduction of MB. In the case of AgNP4, although the amount of silver on the surface was found to be the highest (Table 1), which should be favorable for the reduction of MB, the nanoparticle formation rate may have been too high, resulting in a quick formation and agglomeration of small nuclei. As previously noted, (Figure 1 and Figure 3), the formation of silver nanoparticles for AgNP4 was faster than for either AgNP2 or AgNP3, which likely led to quick nuclei agglomeration; the substantial agglomeration of particles, large average particle size, and wide particle size distribution for AgNP4 were further confirmed by the results of HRTEM/STEM-EDX. As per AgNP2, although its detected amount of silver on the surface is the same as for AgNP3 (Table 1), the XPS data showed that nanoparticles seem to be less stabilized, as indicated by the lower amount of C–C bonds and higher number of oxygen-containing groups than that of AgNP3. More so, just like AgNP4, AgNP2 exhibited a high degree of agglomeration, a wide particle size distribution, and a large average particle size, all of which negatively affected its ability to reduce MB. 

As per AgNP3 samples synthesized at a higher pH of the extract, the removal percentages for these samples were inferior to the sample synthesized at pH 4.5. Interestingly, although AgNP3-8.5 showed the most homogeneous and narrow particle size distribution with the smallest average particle size among the AgNPs studied (Figure 9, Figure 10 and Figure 11), its performance was worse than that of AgNP3 synthesized at pH 4.5 (AgNP3). We believe that the poorer performance of the samples produced at a higher pH could be because the AgNPs are becoming over-capped by the biomolecules of the CNSL extract. A higher pH environment changes the ionization state of the biomolecules, which can lead to their stronger interaction and adsorption onto the surface of AgNPs [42]. In turn, this hinders the catalytic activity of the nanoparticles by blocking their active sites and making them unavailable for MB reduction. Indeed, it can be seen in Table 1 and Figure 7 and Figure 8 that there are more oxygen-containing functionalities and less silver for AgNP3-6.5 and AgNP3-8.5 than for AgNP3 (made at pH 4.5). Therefore, although particles produced at a higher pH exhibit uniformity, small size, and little agglomeration, over-capping is likely the major reason for their inferior performance. Overall, altering the pH of the extract does not seem to be necessary for optimizing the synthesis of AgNPs and for improving their performance towards the reduction of MB. 

The results of this study were compared to some literature studies (Table 3). In this work, on average, 50% removal of MB was achieved within the first 20 min for all the samples studied, but the kinetics of discoloration slightly differed thereafter depending on the sample. The highest % removal was recorded for AgNP3, where some batches showed up to 70–80% removal within the first 20 min (Figure S1) and, on average, about 80% removal within 3h of testing. Considering that catalytic reduction is the main process for MB removal here, these results are impressive and possess a novelty aspect, as the reduction here is achieved in the absence of NaBH_4_ and light, commonly used to facilitate the reduction and degradation of MB, respectively, and simply by using the natural reducing and stabilizing agent—CNSL—that facilitates the electron transfer processes, resulting in MB reduction and high removal percentages.

## 4. Materials and Methods

### 4.1. Preparation of AgNPs 

Cashew nutshells were provided by the Sunshine Nut Company in Boane District, Mozambique [49]. A mixture with a ratio of 1 g of ground shells to 12.5 mL of deionized water was prepared and boiled for 15 min in a round bottom flask on a heating mantle at 100 °C; the mixture was covered with aluminum foil to help prevent evaporation. After, the mixture was cooled to room temperature and filtered to separate the cashew nutshells and the cashew nutshell liquid (CNSL). The separated CNSL is referred to as the extract, and it was freshly prepared for each experiment. 

Silver nitrate was purchased from Sigma-Aldrich^®^ (St. Louis, MO, USA). First, 2–4 mM concentrations of silver nitrate were prepared in 100 mL amber volumetric flasks. Then, 10 mL of the extract and 90 mL of deionized water were pipetted into a 250 mL beaker. Next, 100 mL of the necessary concentration of silver nitrate was added. The beaker was covered and left overnight at room temperature so the synthesis reaction could proceed; the mixture changed from a golden yellow to dark gray, indicating nanoparticle formation. Although left overnight, the synthesis of all the samples was completed within 5–6 h, and no change in the absorption spectra was detected thereafter. Afterward, the reaction mixture was centrifuged using Thermo Sorvall Legend XTR centrifuge at 12,000 rpm for 15 min (Thermo Fisher Scientific, Waltham, MA, USA), the solvent above the AgNPs was decanted, and the isolated AgNPs were dried in air. The resulting AgNPs were named AgNP2, AgNP3, and AgNP4 where the number following the letters corresponds to the millimolar concentrations of silver nitrate used during the synthesis. 

Testing the synthesis at different pH values involved using sodium hydroxide and hydrochloric acid purchased from Sigma-Aldrich^®^. Before the addition of silver nitrate, the 250 mL beaker with 100 mL of diluted extract was placed on a hot plate with a stir bar at 300 rpm. Sodium hydroxide or hydrochloric acid was added dropwise until the pH value reached 6.5 and 8.5, monitored potentiometrically. When the pH reached the desired value, 100 mL of 3 mM silver nitrate was added, and the steps stated above were followed. The resulting AgNPs were named AgNP3-6.5 and AgNP3-8.5, where the numbers following the letters correspond to the millimolar concentration of silver nitrate and pH during the synthesis, respectively. 

**Scheme 1:**
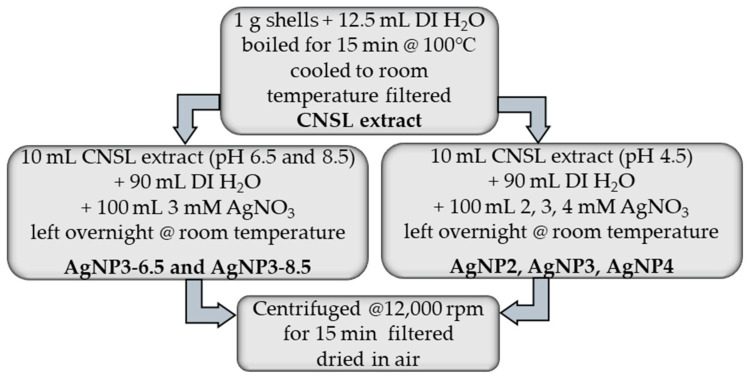
Preparation steps for the synthesis of AgNPs.

The schematic of the synthesis process of AgNPs is presented below (Scheme 1).

### 4.2. Dye Removal Studies

Methylene blue, MB (C_16_H_18_ClN_3_S × H_2_O), was purchased from Sigma-Aldrich^®^. The solution of MB was prepared in a concentration of 20 mg/L. In a typical run, 10 mg of AgNPs were added to 20 mL of the 20 ppm MB solution on a compact digital mini rotator (Thermo Scientific) at 175 rpm. Aliquots of the mixture were collected every 30 min and centrifuged, and the absorbance values were measured at 663 nm, using an Agilent 8453 UV-Visible Spectrophotometer (Agilent Technologies, Santa Clara, CA, USA). The removal studies were conducted in the dark and under green light (Kessil KSPR 160L-525nm LED Photoredox Light, Richmond, CA, USA) for different intervals until equilibrium was reached. The percent removal at any point in time was calculated using Equation (1):

(1) $$\%Removal=\left(c_{0}{-c}_{t}\right)/c_{0}\times 100$$

where $c_{0}$ is the initial concentration of MB and $c_{t}$ is the concentration of MB at time *t*.

### 4.3. X-ray Photoelectron Spectroscopy (XPS)

XPS studies were performed on a Physical Electronic spectrometer (PHI Versa Probe II, Physical Electronics, Chanhassen, MN, USA) using monochromatic Al Kα radiation (25.1 W, 15 kV, 1486.6 eV) and a dual beam charge neutralizer for analyzing the core-level signals of the elements of interest with a hemispherical multichannel detector. The activated carbon sample spectra were recorded with a constant pass energy value of 29.35 eV and a beam diameter of 200 µm. The energy scale was calibrated using Cu 2*p*_3/2_, Ag 3*d*_5/2_, and Au 4*f*_7/2_ photoelectron lines at 932.7, 368.2, and 83.95 eV, respectively. The X-ray photoelectron spectra obtained were analyzed using PHI SmartSoft software 4.3.1 and processed using the MultiPak 9.6.0.15 package. The binding energy values were referenced to the C 1*s* signal at 284.5 eV. Shirley-type background and Gauss–Lorentz curves were used to determine the binding energies. Atomic concentration percentages of the characteristic elements were determined considering the corresponding area sensitivity factor for the different measured spectral regions.

### 4.4. X-ray Diffraction (XRD)

Laboratory X-ray powder diffraction (XRPD) patterns were collected on a PANanalytical EMPYREAN automated diffractometer (Malvern Panalytical, Malvern, UK). Powder patterns were recorded in the Bragg–Brentano reflection configuration by using the PIXcel 3D detector (Malvern Panalytical) with a step size of 0.017° (2*θ*). The powder patterns were recorded between 5 and 80 in 2*θ* with a total measuring time of 30 min.

### 4.5. High-Resolution Transmission Electron Microscopy (HRTEM)/Scanning Transmission Electron Microscopy (STEM)

The particle morphology and its distribution were obtained with HRTEM by a TALOS F200X instrument (Thermo Fisher Scientific, USA), which also operates in STEM mode. The microscope is equipped with a HAADF detector (Thermo Fisher Scientific), working at 200 kV and 200 nA. The microanalysis was carried out with an EDX Super-X system provided with 4 X-ray detectors and an X-FEG beam. Particle size distribution was performed using Image J software, counting at least 400 particles for each sample.

## 5. Conclusions

To summarize, CNSL was successfully utilized to produce silver nanoparticles of different properties. The conditions where the concentration of silver ions was kept at 3 mM and the pH of the extract at 4.5 proved to be the most stable and efficient for the synthesis, producing particles of small size and homogeneous and narrow particle size distribution. Such particles further displayed the best removal properties for MB, with some batches achieving up to 70–80% removal within the first 20 min. Higher concentrations of silver ions significantly increased the rate of nanoparticle formation and the amount of silver in nanoparticles but resulted in substantial particle agglomeration and wide particle size distribution. This, in turn, lowered the ability of these particles to remove MB. Furthermore, the increase in the pH of the extract to 8.5 resulted in an instant nanoparticle formation and produced particles of small size and highly homogeneous and narrow particle size distribution with no observed agglomeration. However, the rate of the synthesis, in this case, may have been too high, leading to unstable synthesis and particles becoming over-capped by the CNSL extract molecules. As a result, nanoparticles synthesized at a higher pH of the extract did not show any improvement in MB removal in comparison to the particles synthesized at a lower pH. Finally, only a small increase in the % removal of MB was observed under visible light compared to in the dark. This suggests that light here plays only a minor role and that it is the catalytic nature of AgNPs related to the presence of the stabilizing and reducing components of CNSL that is responsible for the removal of MB via electron transfer processes leading to MB reduction.

## Figures and Tables

**Table 1:** Results of the deconvolution of the XPS core-level spectra for C 1*s*, O 1*s*, and Ag 3*d*, and the element content in atomic percentage for AgNP samples.

	C 1*s* (%)			O 1*s* (%)	Ag 3*d* (%)
BE, eV	284.8 C–C	286.5 C–O	288.6 O–C=O	532.6 O–C/O=C	368.5/374.5
AgNP2	86.6	10.1	2.3	19.4	1.6
AgNP3	91.5	8.4	ND ^1^	12.3	1.6
AgNP4	78.7	16.3	5.0	20.4	2.9
AgNP3-6.5	85.5	12.4	2.1	16.9	0.4
AgNP3-8.5	78.6	17.1	4.3	19.0	0.8

^1^ “Not Detected”.

**Table 2:** Lower and upper ranges of silver particle size for each sample.

Sample	AgNP2	AgNP3	AgNP4	AgNP3-6.5	AgNP3-8.5
Particle size range (nm)	6.2–25.6	0.2–13.2	4.6–27.2	2.1–17.7	3.8–9.8

**Table 3:** Comparison of the results of this study to the literature.

Plant Extract	Light Source	AgNP Size (nm)	Time (h)	% Removal	Ref.
Cashew nutshell liquid	Green	0.2–13.2	3	81	This study
	Dark		3	78	
Camellia sinensis leaf	Solar	25–40	72	95	[36]
Morinda tinctoria leaf	Solar	79–96	72	95	[11]
Ulva lactuca seaweed	Solar	49	12	85	[48]
Kitchen vegetable waste	Solar	10–100	3	88	[38]
Indian screw tree	Solar	25–45	0.5	95	[40]

## Data Availability

Data are contained within the article and Supplementary Materials.

## Supplementary Materials

The following supporting information can be downloaded at: https://www.mdpi.com/article/10.3390/molecules29163895/s1, Figure S1: The MB discoloration curves shown are for all the batches of the AgNP samples studied.
