# Supplementary figures and images for: Green Synthesis of Silver Nanoparticles Using Cashew Nutshell Liquid (CNSL): Characterization and Methylene Blue Removal Studies
Source: Molecules. 2024 Aug 17;29(16):3895. doi: 10.3390/molecules29163895 (PMC11357457; doi:10.3390/molecules29163895)

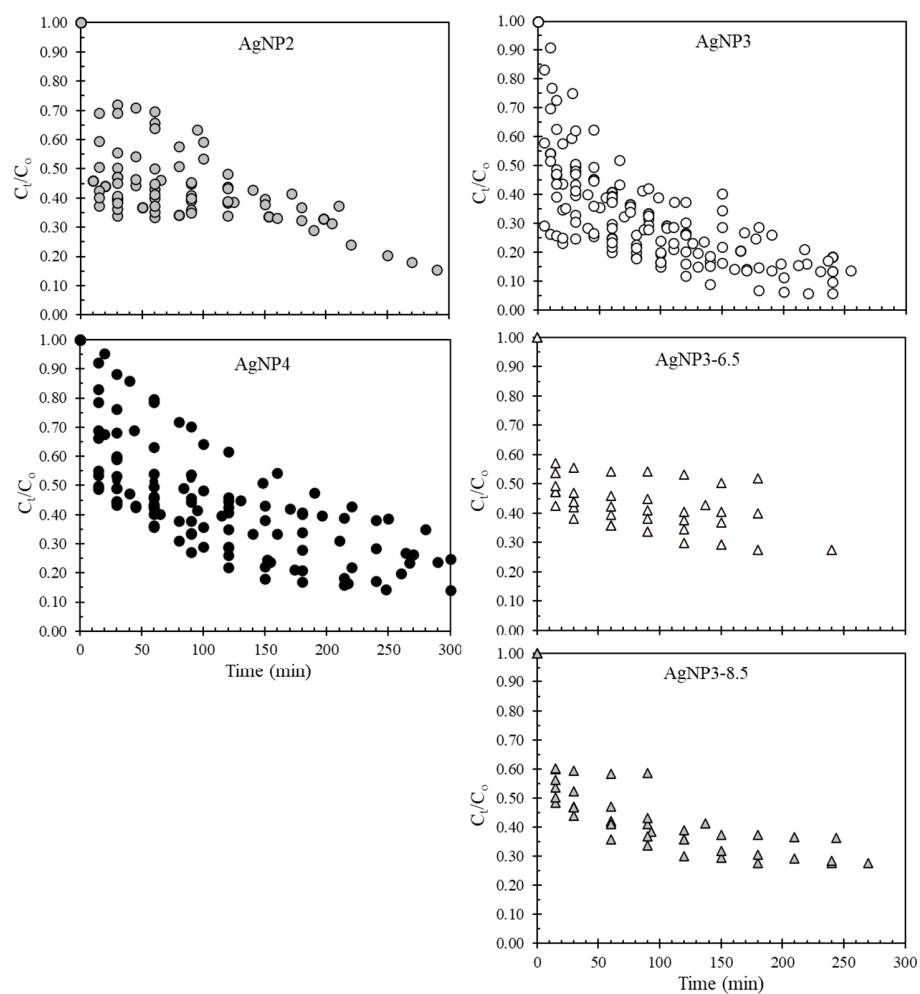

Figure S1. The MB discoloration curves shown for all the batches of the AgNP samples studied.

Supplement: Supplementary file 1 [file molecules-29-03895-s001.zip › molecules-3083482-supplementary.pdf]
